# Supplementary material for: Risk of Esophageal and Gastric Cancer in Patients with Type 2 Diabetes Receiving Glucagon-like Peptide-1 Receptor Agonists (GLP-1 RAs): A National Analysis
Source: Cancers (Basel). 2024 Sep 22;16(18):3224. doi: 10.3390/cancers16183224 (PMC11430483; doi:10.3390/cancers16183224)
Supplement: Supplementary file 1 [file cancers-16-03224-s001.zip › cancers-3179406-supplementary.pdf]

ICD-10 Codes used in analysis:

Criterion
Type 2 Diabetes Mellitus (E11)
Family history of malignant neoplasm of digestive organs (Z80.0)
Infectious mononucleosis (B27)
Chronic atrophic gastritis (K29.4)
Vitamin B12 deficiency anemia due to intrinsic factor deficiency (D51.0)
Helicobacter pylori as the cause of diseases classified elsewhere (B96.81)
Gastro-esophageal reflux disease (K21)
Unspecified chronic gastritis (K29.5)
Malignant neoplasm of stomach (ICD10CM:C16)
